# Assessing the speed of individual bacteria dispersing on mycelial networks

**DOI:** 10.1007/s10682-025-10329-4

**Published:** 2025-01-25

**Authors:** Matteo Buffi, Thierry Kuhn, Diego Gonzalez, Saskia Bindschedler, Patrick S. Chain, Xiang-Yi Li Richter, Pilar Junier

**Affiliations:** 1 Laboratory of Microbiology, University of Neuchâtel, Rue Emile-Argand 11, 2000 Neuchâtel, Switzerland; 2 Laboratory of Eco-Ethology, University of Neuchâtel, Rue Emile-Argand 11, 2000 Neuchâtel, Switzerland; 3 Bioscience Division, Los Alamos National Laboratory, P.O. Box 1663, Los Alamos, NM 87545 USA; 4 Institute of Ecology and Evolution, University of Bern, Baltzerstrasse 6, 3012 Bern, Switzerland; 5 Department of Biology, University of Konstanz, Universitaetsstrasse 10, 78464 Constance, Germany

**Keywords:** *Pseudomonas putida*, *Pythium ultimum*, Fungal highways

## Abstract

The movement of bacteria on the hyphae of fungi and other mycelial-forming organisms is an important process that determines their ability to actively disperse in water-unsaturated habitats. However, direct observation and characterization of bacterial cell movement on mycelial networks have been difficult to achieve. In this study, we developed a new method that uses high-speed video recording to track the dispersal of individual fluorescently tagged cells of two closely related strains of *Pseudomonas putida* (UWC1 and KT2440) over the mycelial network of the oomycete *Pythium ultimum*. We found high intra-population heterogeneity and between-population differences in dispersal speeds for the two bacterial strains. The fitting of the speed distribution functions led to the separation of speeds into two ranges (fast/slow) at an intersection of the fitted curves. In the lower speed range, the UWC1 strain dispersed faster, while the KT2440 strain moved faster in the higher speed range. This finding helps explain conflicting competition outcomes revealed in previous studies and suggests that population mean speed alone does not capture key aspects of bacterial dispersal in mycelial networks. Our new method opens the possibility of studying bacterial dispersal, competition, and other social interactions in spatially heterogeneous environments, such as soils.

## Introduction

The dispersal of bacteria plays an important role in ecosystem processes such as nutrient cycling, bioremediation of pollutants, or the biorecovery of valuable materials (Kohlmeier et al. 2005; Banitz et al. 2013; Losa and Bindschedler 2018; Custer et al. 2022). While fundamental knowledge regarding the mechanisms of bacterial motility is well-established (Kearns 2010), particularly in controlled laboratory conditions, there is still much to learn about the complexity of bacterial movement in natural ecosystems, especially spatially heterogeneous environments that are not saturated with water. In liquid media, where the majority of the studies have been performed, most bacteria use one or more rotatory flagella to swim. In other media, such as hydrated substrates, bacteria undergo hyperflagellation (increase in the number of flagella) and move collectively during swarming. Alternatively, other structures such as Type IV pili have been involved in twitching, especially on solid surfaces, while adhesins, located on the surface of the cells, have been involved in gliding motility (Wadhwa and Berg 2021). Regardless of the type of motility, one common aspect that limits bacterial dispersal in the environment is the level of water saturation or the presence of air-filled gaps (Or et al. 2007). A strategy that has been shown to allow bacteria to overcome dispersal limitation in spatially heterogeneous environments that are not saturated with water such as soils (Simon et al. 2017; Junier et al. 2021) or cheese rinds (Zhang et al. 2018) is the active movement of bacteria in the liquid film formed around hyphae of hyphae-forming organisms such as filamentous fungi or Oomycetes. The hyphae constitute in this way a network of “fungal highways” that serve as a physical pathway that bacteria can use to colonize areas that would be otherwise inaccessible (Kohlmeier et al. 2005; Simon et al. 2015). Fungi have also been shown to benefit from allowing bacteria to move on their hyphae by increasing their uptake of essential nutrients (Lohberger et al. 2019; Jiang et al. 2021). Fungal highways have been shown to be highly relevant for soil ecological functioning (Wick et al. 2007; Simon et al. 2017; Junier et al. 2021) or in processes such as food production (Zhang et al. 2018).

Other than the need for flagella (Pion et al. 2013) or the “hitchhiking” of non-flagellated bacteria on other flagellated bacteria (Warmink et al. 2011; Kuhn et al. 2022b) many aspects of fungal highways remain understudied. One of those is the level of control that hyphae-forming microorganisms exert on the bacteria moving on their hyphae. For instance, the thickness of the liquid film on the surface of hyphae is thought to restrict the free movement of individual cells (Or et al. 2007). This parameter depends not only on environmental conditions (Wong and Griffin 1976), but also varies among different taxa based on their ability to produce different surfactants or to modify the composition of the cell wall (Kohlmeier et al. 2005; Gow et al. 2017). Consequently, bacteria using hyphae to disperse may encounter areas with varying degrees of “crowding” or “traffic jams”, influenced by the intrinsic properties of the species supporting dispersal, the environmental conditions, or a combination of both. Under these conditions, bacteria might adopt different strategies to overcome these limitations. Adaptations allowing the modification of speed, such as expressing different numbers of grappling-hook-like pili, have been observed in previous studies as a strategy to overcome crowding in *Pseudomonas aeruginosa* in collective movement across surfaces (Meacock et al. 2020). Given the complex spatial structure and diverse spatial niches in fungal highways, we could expect a bet-hedging strategy that favors plastic movement speeds to better use exploration and exploitation opportunities in the dispersal networks. We hypothesized that this adaptation applies to bacteria moving on fungal highways, but to test this, the speed of individual cells on fungal highways need to be measured. Accomplishing this task is challenging because it requires an experimental system where the movement of individual bacteria can be observed with sufficient magnification and temporal resolution to enable accurate tracking of individual cells.

Therefore, in this study, we present an approach to observe the trajectory and measure the speed of individual cells moving in the liquid film surrounding hyphae. For this, we combine a recently developed method called the “fungal drops” that allows for establishing fungal highways at the laboratory scale (Buffi et al. 2023) with high-speed imaging and video analysis to achieve tracking of individual bacterial cells. Although this system does not fully capture the complexity and heterogeneity of real soil, it provides a feasible environment for measuring the movement of single cells in dispersal networks generated by mycelial-forming organisms. This is challenging to achieve in other systems used to model fungal highways, such as water-saturated microfluidic devices or agar cultures, as both hold continuous water films where bacteria can disperse independently from mycelial network (Pion et al. 2013; Buffi et al. 2023). Furthermore, as bacterial behavior can be heterogeneous (Xue et al. 2009), a detailed examination of the variation in speeds must be accomplished by measuring the distribution of instantaneous movement speeds of individual bacterial cells within a population. This approach provides insights into the strategies adopted by bacteria dispersing in spatially complex environments, such as mycelial networks in the soil, as such environments may select for bet-hedging strategies for exploring and exploiting different spatial niches.

## Materials and methods

### Preparation of mycelial suspensions

The filamentous oomycete *P. ultimum* was obtained from the microbial collection of the laboratory of microbiology, University of Neuchâtel, Switzerland, and was maintained on Malt Extract Agar (MEA, 12 g/l malt extract [SIOS, Homebrew], 15 g/l Agar [Biolife]). For the preparation of mycelial suspensions, *P. ultimum* was incubated in M9 minimal liquid medium at room temperature and under constant agitation (Lab Shaker, Adolf Kühner AG) at 120 rpm for 5 days. The mycelium was then fragmented and homogenized using an ULTRA-TURRAX® (IKA® T18 basic) at max speed for 10 s and then washed 3 times with physiological saline solution (0.9% NaCl). The resulting hyphal suspension was quantified with a Neubauer chamber (BIOSYSTEMS® 0.01 mm) and then resuspended at a final concentration of circa 10 hyphal fragments/µl in a M9 minimal liquid medium (64 g/l Na_2_HPO_4_•7H_2_O, 15 g/lKH_2_PO_4_ 2.5 g/l NaCl, 5.0 g/l NH_4_Cl).

### Preparation of the bacterial inoculum

We used two closely related *Pseudomonas putida* strains deposited in the bacterial collection of the laboratory of microbiology, University of Neuchâtel, Switzerland: *P. putida* KT2440 and *P. putida* UWC1. Both strains are flagellated and have originally been isolated from a rhizospheric soil (Regenhardt et al. 2002). The two strains are constitutively tagged with fluorescent proteins via mini-Tn7 transposon insertions, KT2440 with a green fluorescent protein (GFP) and UWC1 with mCherry (Rochat et al. 2010; Dechesne et al. 2010). The different tags allowed us to identify the strains using epifluorescence microscopy. Bacterial suspensions were obtained from overnight cultures incubated in Nutrient Broth (NB, 25 g/L, Carl Roth, AE92.2) at room temperature and under constant agitation (120 rpm). The cells were then collected by centrifuging (5000 g for 5 min), washed 3 times in physiological saline solution, and then resuspended in M9 minimal liquid medium at an optical density of 1, corresponding to approximately 10^9^ cells/ml.

### Glass slides treatment

**Fig. 1:**
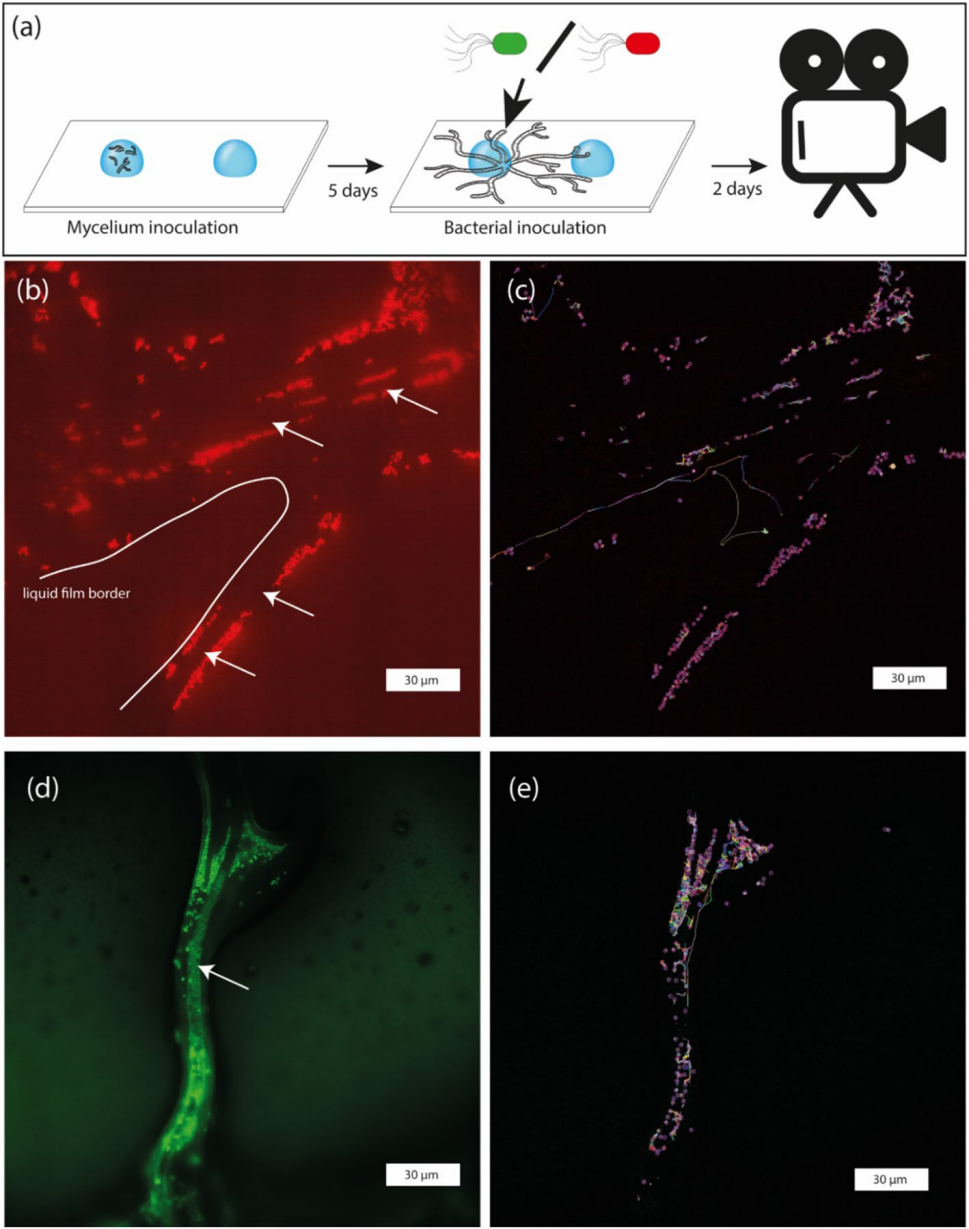
Tracking the movement of bacteria in the *Pythium ultimum* mycelial network. Panel **a**: overview of the inoculation of drops with *P. ultimum* forming a mycelial network connecting the two drops; after 5 days post inoculum one of the two bacterial strains was added; bacteria moving along the hyphae network between the drops were filmed after 2 days. Panels **b** and **d**: snapshots of the microscopic videos of the liquid film around *P. ultimum* hyphae. Panels **c**, **e**: snapshots of the microscopic videos of bacterial movement on fungal hyphae. Panels **b** and **d** show the fluorescently labelled UWC1 (red) and KT2440 (green) strains of *Pseudomonas putida*, respectively. Panels **c** and **e** show the corresponding tracking results, where the magenta circles represent individual cells, and the coloured lines represent movement trajectories. The corresponding videos are provided in the Zenodo repository: https://zenodo.org/records/14163816; the frame-wise tracking trajectories are provided in the Zenodo repository: https://zenodo.org/records/14166198

The “fungal drops” (Buffi et al. 2023) system was set on a rectangular (24 × 60 mm) glass cover slide with thickness #0 (0.08–0.130 mm; see Fig. 1). To foster *P. ultimum* growth and the formation of the liquid film associated with its hyphal network, the cover slides were treated with Poly-D-Lysine (0.1 mg/ml; GIBCO^®^) as follows. First, the cover slides were immersed in 37% HCl (Sigma ref.: 84,422) overnight, then thoroughly rinsed, and autoclaved in milli-Q H_2_O. After this cleaning step, the cover slides were coated following the product manual (GIBCO^®^) and used once dried. When not used directly, the slides were stored at 4 °C and used in the coming weeks.

### Inoculation

Two 15 µl drops were placed on the coated cover slide at a distance of 0.5 cm from each other. One of the drops consisted of the initial inoculum drop and contained the hyphal suspension, while the other contained sterile M9 minimal medium. The inoculated “fungal drops” systems were then incubated at room temperature in a humid chamber consisting of a glass bell containing moistened Vermiculite at the bottom (Buffi et al. 2023). *P. ultimum* was left to grow over five days until its network connected the two drops. At five days post inoculation (dpi), 2 µl of the bacterial suspension was added to the inoculum drop previously inoculated with *P. ultimum*. The experiments were performed in 5 replicates for each bacterial strain.

### Recording

To observe and record bacteria moving along fungal highways, an inverted Nikon Ti2-E microscope equipped with an OkoLab box incubator to control relative humidity at > 80% and temperature at 24 °C was used. All observations were done using an oil immersed 40X objective at a numerical aperture (NA) of 1.45, a working distance (WD) of 0.13 mm, a Plan Apo lambda DIC (differential interference contrast) objective lens, a color camera (Orca Fusion BT 2304 × 2304 pixels, 6.5 μm × 6.5 μm), and a spectra III light engine (8-wavelength; GFP: 474/27 nm; mCherry-like: 578/21 nm). We ran the microscope in time-lapse imaging mode, with a frame rate acquisition speed of 0.024 s. The field of view was set to the mycelial network connecting the drops. The pictures obtained were then used to compile a video using the software Image J (Schneider et al. 2012). A total of 5 videos for each bacterial strain were produced and analyzed (details see next section).

### Tracking

To track bacterial movement in the liquid films of the mycelial network of *P. ultimum*, we used the plugin TrackMate (Tinevez et al. 2017) of the software Fiji (version 1.8.0), which allows for the automated tracking of individual bacterial cells. TrackMate creates a file with the coordinates of each cell at any time point. The tracking algorithm identified some very fast cells, which were suspected to be an artifact (e.g., a cell moving out of the focal plane followed by another one moving in). Those were excluded by manually checking the videos. After manual checking, the fastest instant speed of KT2440 and UWC1 cells was around 115 um/s. To be more stringent in the inclusion criteria and to avoid tracking artifacts, we set an upper cutoff of the speed at 100 um/s. This cutoff excluded 1.137% of the UWC1 speed measurement (Supplementary Information Appendix Section 1) and 0.741% of the KT2440 cells (Supplementary Information Appendix Section 2). We further excluded tracking trajectories shorter than 5 µm in distance from the analysis because they mainly correspond to cells inside mini-colonies. The movements were caused by cells pushing each other to rearrange positions, which does not lead to dispersal. We also excluded tracking trajectories shorter than 0.25 s because they correspond to cells entering the focal plane only transiently. The trajectories of these cells were often not parallel to the focal plane, which can cause errors when calculating their movement speeds. After cleaning up the data, we used the statistical software R (R Core Team (2022) to calculate the movement speed of cells by dividing the distance between a cell’s coordinates in two consecutive frames with the time difference between the frames.

### Statistical analysis

We fitted the speed distribution data recorded for the two bacterial strains to simple forms of continuous probability distributions and evaluated their goodness of fit based on a series of evaluation criteria, including the Bayesian information criterion, Akaike information criterion, Hannan-Quinn information criterion, and the Log-likelihood. The top ten best fits for each strain and their corresponding goodness of fit evaluation results are provided in Supplementary Information Appendix Section 3. We then separated the speed distributions arbitrarily into a slow and a fast movement range at an intersection of the fitted probability distribution curves. In addition, we performed the non-parametric Kolmogorov–Smirnov Test to compare the speed distributions and the Kruskal–Wallis tests to compare the mean speed between the KT2440 and UWC1 strains in the slow and fast movement ranges. All statistical analyses and plots were performed using the software Mathematica. The Notebook code is provided in the Supplementary Information.

## Results

To establish a mycelial network for the dispersal of bacteria, we first grew the oomycete *P. ultimum* in a “fungal drop” system (Buffi et al. 2023) adapted for high-resolution microscopy. The main adaptation from the original method consisted of the use of ultra-thin coated cover slides instead of treated Petri dishes to enable tracking of individual bacteria cells moving on the liquid film surrounding the hyphae directly in the specific setting of the microscope (mostly regarding focal distance). The oomycete grew into a filamentous hyphal network and connected the two drops after 5 days (Fig. 1a) in all 5 replicates. Two *P. putida* strains, each with a distinct fluorescent tag (KT2440-GFP and UWC1-mCherry), were used to assess the movement of bacteria in the mycelial network. The two strains were able to grow and effectively used the liquid film surrounding the mycelial network of *P. ultimum* to disperse between the two drops (Fig. 1b–d). The fluorescence signals were detected clearly for both the GFP-tagged (Fig. 1b) and mCherry-tagged strains (Fig. 1d), allowing for accurate tracking and speed measurement for the two strains (Fig. 1c, e).

**Fig. 2:**
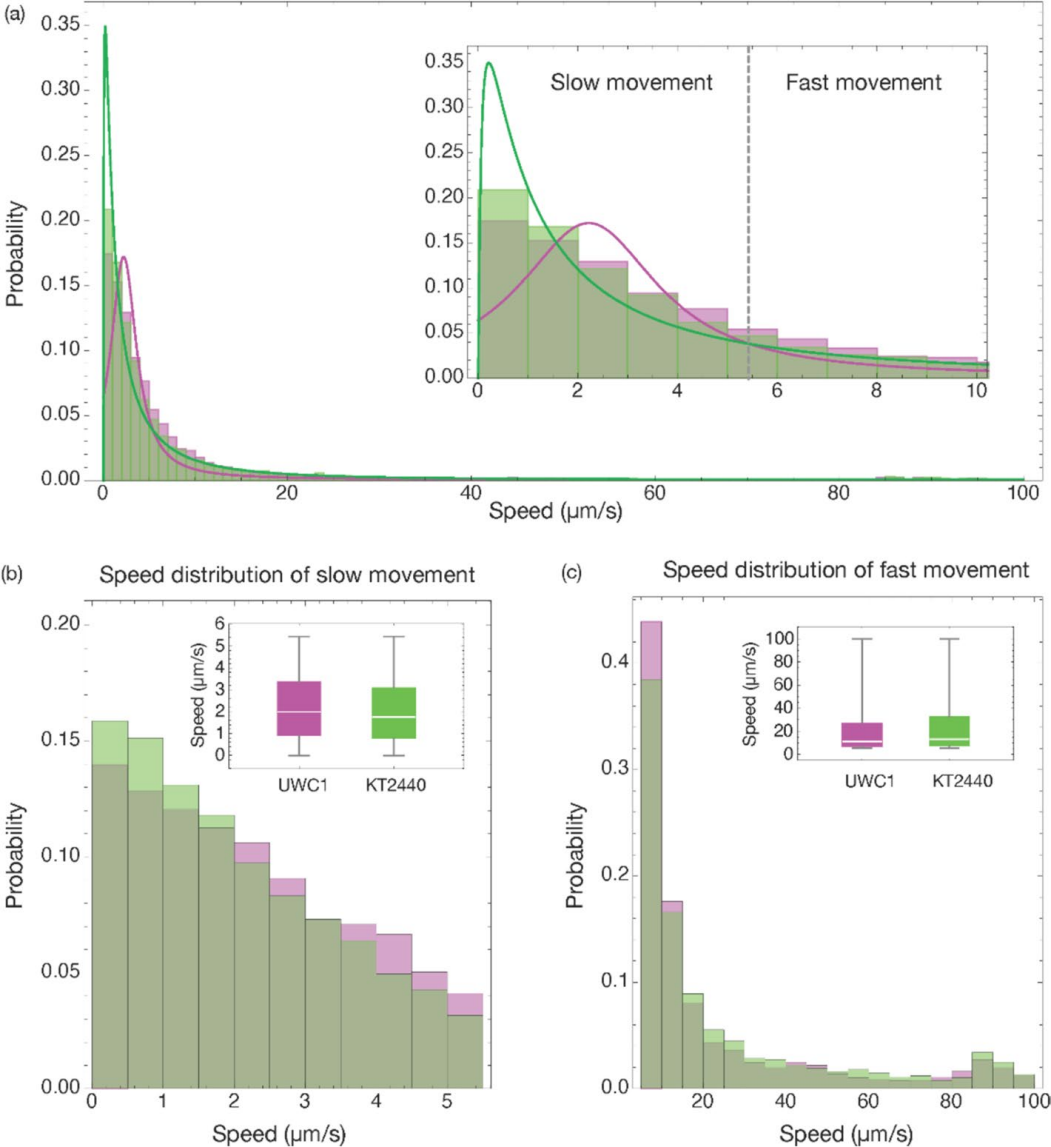
Speed of movement distribution analysis. **a** Speed distribution of the UWC1 (magenta) and KT2440 (green) cells. The corresponding green and magenta lines are fitted probability distributions. The inset shows a zoomed-in view of the speed distribution below 10 µm/s. We separated the cell speed into slow and fast movement ranges at an intersection of the fitted distribution curves. **b** Speed distributions in the slow movement range; UWC1 cells generally move faster than the KT2440 cells. **c** Speed distributions in the fast movement range; KT2440 cells generally move faster than the UWC1 cells. Details on the best-fitting models and goodness-of-fit analyses are provided in the Supplementary Information provided in the Zenodo repository: https://zenodo.org/records/14208231, Appendix Section 3. For the separate representations of the histograms and the fitted probability distribution curves please see Supplementary Information Appendix Sect. 5

Because cells were not individually labeled, once they moved in or out of the focal plane, it was impossible to determine if the cells observed corresponded to the same cell or a new one. Therefore, we use the number of independent measurements instead of the number of cells to evaluate the speed distributions of different populations (Table 1). The range of measured speeds for the moving cells of the two strains was similar, from slow-moving (< 1 µm/s) up to over 100 µm/s (Fig. 2a). We fitted the cell movement speed data to different probability distribution functions and evaluated their goodness of fit. The shapes of the speed distribution patterns for the two examined strains were similar in the sense that around 2/3 of the cells moved at relatively low speeds (< 5 µm/s), while a small proportion of cells moved much faster. However, the speed distributions of the two strains had significantly different statistical features. The speed distribution of KT2440 cells (GFP-tagged, hence green) exhibited the best fit to a mixture of a lognormal distribution and a uniform distribution (Fig. 2a, green line; Supplementary Information Section 3). In contrast, the speed distribution of UWC1 cells (mCherry-tagged, hence red) fitted best to a mixture of a Cauchy distribution and a uniform distribution (Fig. 2a, magenta line; Supplementary Information Section 3). More specifically, the difference in distribution meant that a larger fraction of the cells for the KT2440 strain were faster, while cells from strain UWC1 were faster at lower speeds. To test whether the mixed distributions were due to intrinsic features of the data, we split the dataset for each strain at an arbitrarily chosen 60 µm/s into slow and fast subpopulations and fitted each subset of data to probability distribution functions. The top three best-fitting functions for each data subset were still mixed functions, suggesting that the mixed distributions were intrinsic features of the datasets (Supplementary Information Section 4).

**Table 1:** Datasets included in the speed of movement distribution analysis for the UWC1 and KT2240 strains

UWC1	Number of independent speed measurements	Mean slow cells speed (um/s)	Mean fast cells speed (um/s)
rfp_2days022_11fps_2_movement.csv	1476	2.32 ± 1.51	22.34 ± 23.26
rfp_2days025_11fps_2_movement.csv	1473	2.05 ± 1.47	23.24 ± 23.11
rfp_2days026_11fps_2_movement.csv	1501	2.31 ± 1.49	24.56 ± 26.30
rfp_2days028_11fps_2_movement.csv	1463	2.16 ± 1.47	24.54 ± 27.51
rfp_2days031_11fps_2_movement.csv	1211	2.18 ± 1.49	19.21 ± 22.47
Combined data for UWC1	7124	2.20 ± 1.49	22.81 ± 24.54

KT2440	Number of independent speed measurements	Mean slow cells speed (um/s)	Mean fast cells speed (um/s)
gfp_2days002_11fps_2_movement.csv	741	1.54 ± 1.10	42.28 ± 36.60
gfp_2days004_11fps_2_movement.csv	1572	2.03 ± 1.44	24.20 ± 25.81
gfp_2days005_11fps_2_movement.csv	1315	2.04 ± 1.42	27.09 ± 27.88
gfp_2days011_11fps_2_movement.csv	1781	2.28 ± 1.54	24.82 ± 22.06
gfp_2days015_11fps_2_movement.csv	935	2.18 ± 1.50	22.69 ± 29.39
Combined data for KT2440	6344	2.02 ± 1.44	25.06 ± 25.40

The raw data is included in Supplementary Information provided in the Zenodo repository: https://zenodo.org/records/14208231. As cells could not be tracked individually when moving out of focus, we counted the number of individual measurements (N) instead

Considering the between-individual variation of speed distribution within each strain and the statistically different distributions between the strains, we attempted to compare the movement speed patterns by establishing two categories of movement speed ranges. For this, we separated the speed distributions arbitrarily into a slow and a fast movement range at an intersection of the fitted probability distribution curves (Fig. 2a, inset) at 5.44 µm/s. In the fast range, KT2440 cells moved faster (mean speed 25.06 µm/s) than UWC1 cells (22.81 µm/s) (Kruskal–Wallis-test *p*-value = 1.09e-4). In contrast, in the slow range, UWC1 cells moved faster (mean speed 2.20 µm/s) than KT2440 cells (mean speed 2.02 µm/s) (Kruskal–Wallis-test *p*-value = 2.86e-8).

## Discussion

This study represents, to the best of our knowledge, the first successful measurement of speed distributions of individual bacterial cells dispersing on mycelial networks. This was made possible by combining a previously developed method (i.e., fungal drops) designed to better visualize mycelium at the microscale (Buffi et al. 2023) and the use of an inverted widefield fluorescence microscope with an incubation chamber and a high-speed camera. With this approach, we were able to perform the tracking and measurement of the speed of single cells. One interesting result is the maximum speed at which bacteria moved on hyphae. Previous research showed that the speed of different bacterial species during swimming or swarming varies greatly from 15 µm/s for *Escherichia coli* (Mitchell et al. 1995) to more than 100 µm/s (max instantaneous movement up to 500 µm/s) in magnetotactic cocci (Bente et al. 2020). One of the bacterial strains we used in this study, *P. putida* KT2440, has been measured to move at an average speed of 20.9 µm/s and reach a peak velocity of 51.2 µm/s in liquid culture during the exponential growth phase (Davis et al. 2011). However, the speed of individual bacteria dispersing on mycelial networks has not been measured previously. Our results showed that cells from both *P. putida* strains displayed great heterogeneity in their movement speeds in the complex environment of mycelial network compared to the simpler environment of liquid culture. Both strains contained static and very slow-moving cells (mainly in micro-colonies), while a small fraction of cells reached a maximum instantaneous movement speed of up to 100 µm/s (mainly along hyphae connecting distant micro-colonies), which is among the fastest movement speeds described so far. It would be interesting to test whether local cell density influences cell movement speeds in future studies with explicit control for this parameter.

Moreover, our study shed light on how intra-population speed variation may influence the competition outcome between the strains. In previous studies assessing spatial competition between the two *P. putida* strains, conflicting results were obtained under different experimental conditions. In the first study (Kuhn et al. 2022b), the two strains competed on hyphal networks formed on an agar plate, where the UWC1 strain had an advantage and often competitively excluded the other strain. In the second study (Kuhn et al. 2022a), the two strains competed in liquid films formed on an abiotic surface (i.e., the “bacterial bridge” device). In the latter case, the KT2440 strain had an advantage and colonized a distant habitat more efficiently. In both studies, only the bulk dispersal capability of the populations was assessed. Results obtained in the current study suggest that within-strain heterogeneity of dispersal speed can help reconcile those contradicting outcomes. A higher mean speed in the slower range, where a larger fraction of the population was found (67.41% of KT2440 and 65.49% of UWC1), might explain the advantage of the UWC1 strain in hyphae networks on an agar plate, where the movement speed of cells was limited by the low level of water saturation (Kuhn et al. 2022b). By contrast, a higher mean speed in the faster range of the KT2440 strain may have resulted in its competitive advantage on the abiotic surface, where the degree of water saturation was much higher than in the hyphal networks in an agar plate (Kuhn et al. 2022a). The heterogeneity in the speeds of individual cells suggests that the population mean speed does not reflect the dispersal of individual cells in spatially complex environments, such as soils (Choudoir and DeAngelis 2022) and fermented foods (Louw et al. 2023). We thus speculate that individual variation of dispersal speed serves as a bet-hedging strategy for exploiting diverse spatial niches in complex networks formed by fungi and other mycelial-forming organisms. This hypothesis remains to be tested experimentally, for example, by performing direct competition assays in controlled environments with varying degrees of water saturation, which can be achieved by varying the composition of the media.

By directly measuring the dispersal speed distributions of bacterial cells on hyphal networks, the approach presented here will further our understanding of the characteristics that make bacteria suited to explore spatially complex environments such as soils. For example, it can help us find out if individual cells of the same or closely related bacterial species are equally efficient in using mycelial networks to disperse. The results obtained here suggest this was not the case for the two *P. putida* strains, given their distinct characteristic movement speed distributions under the conditions tested. The fitted probability distribution functions provided a useful statistical description of the heterogeneity in movement speeds, but they revealed no information about the mechanisms causing the difference between the two strains, which remain to be investigated. The heterogeneity of the mycelial network and the resulting liquid paths may select for specific dispersal strategies. Although this has not been investigated in fungal highways, experiments with swarming colonies of *P. aeruginosa* showed that morphological adaptations can influence dispersal success under different degrees of crowdedness. Cells that were genetically modified to express a larger number of pili spread faster at the less crowded colony margins, while cells with fewer pili moved faster under higher cell density (Meacock et al. 2020). A similar diversification of morphological adaptations (with or without a genetic basis) between different species or even within strains of the same species could occur in bacteria moving along fungal highways and other biotic dispersal networks. The mechanisms explaining speed adaptation seem to be largely unexplored. Nevertheless, the flagellum has been suggested to participate in surface sensing in bacteria (Laventie and Jenal 2020), which may help them adjust movement speed according to different surface conditions in the environment. For instance, studies performed in the aquatic bacterium *Vibrio parahaemolyticus* suggested that changes in the rotation speed of flagella upon contact with a surface trigger the expression of lateral flagella for swarming (McCarter et al. 1988). In addition, signaling via c-di-GMP has also been suggested to participate in surface adaptation (Laventie and Jenal 2020). Similar mechanisms might play a role in the speed adaptation in mycelial networks, but this needs to be investigated.

The transport of metabolically resting cells or unrelated microbial species in mono- and multispecies associations within swarming colonies has been considered as a bet-hedging strategy for the colonization of novel habitats under variable environmental conditions (Inghama et al. 2011; Hagai et al. 2014; Finkelshtein et al. 2015). A recent review highlighted the importance of bet-hedging strategies in several categories of microbial lifestyles, including dormancy, resource exploitation, and signaling (Grimbergen et al. 2015). However, the role of speed adaptation as a bet-hedging strategy has not yet been considered as a mechanism for adapting to limited dispersal opportunities in heterogeneous environments. Our method can facilitate future studies addressing this question in complex environments generated by mycelial-forming organisms. For example, by testing whether parameters such as nutrient availability or species composition can affect the features of the dispersal network (e.g., thickness and composition of the liquid film), which further influences the choice of dispersal strategies. Likewise, hitchhiking dispersal (i.e., non-motile bacteria using motile ones to disperse) is another area of research that can benefit from direct observations and assessment of bacterial movement along fungal highways and other biotic networks (Warmink et al. 2011; Kuhn et al. 2022b). Our new method can facilitate such research by imaging and tracking interacting cells labeled with different fluorescence markers simultaneously. Understanding the causes and consequences of such bet-hedging strategies will prove useful for choosing suitable microbial strains for real-world applications, ranging from biotechnology to ecosystem restoration, plant growth promotion, and engineering (Frey-Klett et al. 2011; Krüger et al. 2019; Steffan et al. 2020; Jiang et al. 2021).

## Data Availability

A supplementary appendix file that includes all supporting materials and links to raw data is provided in the Zenodo repository: https://zenodo.org/records/14208231.
